# Improving Collective Estimations Using Resistance to Social Influence

**DOI:** 10.1371/journal.pcbi.1004594

**Published:** 2015-11-13

**Authors:** Gabriel Madirolas, Gonzalo G. de Polavieja

**Affiliations:** 1 Instituto Cajal, Consejo Superior de Investigaciones Científicas, Madrid, Spain; 2 Champalimaud Neuroscience Programme, Champalimaud Center for the Unknown, Lisbon, Portugal; Imperial College London, UNITED KINGDOM

## Abstract

Groups can make precise collective estimations in cases like the weight of an object or the number of items in a volume. However, in others tasks, for example those requiring memory or mental calculation, subjects often give estimations with large deviations from factual values. Allowing members of the group to communicate their estimations has the additional perverse effect of shifting individual estimations even closer to the biased collective estimation. Here we show that this negative effect of social interactions can be turned into a method to improve collective estimations. We first obtained a statistical model of how humans change their estimation when receiving the estimates made by other individuals. We confirmed using existing experimental data its prediction that individuals use the weighted geometric mean of private and social estimations. We then used this result and the fact that each individual uses a different value of the social weight to devise a method that extracts the subgroups resisting social influence. We found that these subgroups of individuals resisting social influence can make very large improvements in group estimations. This is in contrast to methods using the confidence that each individual declares, for which we find no improvement in group estimations. Also, our proposed method does not need to use historical data to weight individuals by performance. These results show the benefits of using the individual characteristics of the members in a group to better extract collective wisdom.

## Author Summary

We modelled how humans interact, and used the models to find strategies that can make groups more accurate. Each individual in a group combines private and public information to make estimations. But when the public information is biased, social information has the effect of making groups agree even more on an incorrect collective estimation. We reasoned that not all individuals should be influenced equally by the incorrect public information. We obtained a model to understand how private and social information are combined, and used it to obtain a value of social resistance for each individual. We then used these values of social resistance obtained from the model to extract the subgroup of people resisting social influence, and found that they give an improved collective estimation. Collective intelligence is thus maximal when taking into account individuality in human behavior.

## Introduction

Francis Galton was the first to experimentally demonstrate the advantages of collective estimations [1]. At a farmers’ fair, he found that the median of the independent estimations made by 784 farmers of the weight of a slaughtered ox was better than any of their individual estimations. Since then, collective estimations, computed as mean, median or geometric mean values of the group, have been shown to improve upon the estimations of most individuals of a group in several different contexts, an effect popularly known as *wisdom of crowds* (WOC) [2–8]. However, human crowds can also be notoriously bad at making collective estimations for many estimation tasks [7, 9]. Social interactions can have an additional negative effect in biased crowds [8, 9]. When individuals learn the estimations of the other members of the group, they typically change their own estimation towards the more common values. After social influence, the collective has thus a distribution of estimations more strongly peaked around the biased solution. This can give the collective perception of an agreement but the value agreed upon can be far from the truth [9].

We propose to turn the negative effect of social interactions to our advantage and improve collective estimations. We do so by taking into account the individuality of the members of the group. Francis Galton argued for each individual counting the same in the collective estimation [1]. But for situations in which most individuals are strongly biased, we would be in a better position with methods selecting the unbiased individuals. Of course, this can be done by finding how well each individual performs in a domain of knowledge and weight them accordingly for similar tasks [10–12].

Here we do not consider the case of access to a classification of individuals by performance. Instead we used the impact of social interactions on estimations to extract individuals in the following way. We first obtained a model of estimation in a collective and used it to measure how much each individual of the collective resists social influence. We tested the model by reanalyzing a dataset in which subjects made estimations before and after social influence [9]. This is a rich dataset that can be used as a reference to test models of social influence [13]. In these experiments subjects were asked to privately estimate the answer to six questions [9]: ‘What is the length of the border between Switzerland and Italy in kilometers?’, ‘How many rapes were officially registered in Switzerland in 2006?’, ‘How many assaults were officially registered in Switzerland in 2006?’, ‘What is the population density of Switzerland in inhabitants per square kilometer?’, ‘How many murders were officially registered in Switzerland in 2006?’ and ‘How many more inhabitants did Zurich gain in 2006?’ After their private estimation for each question, each subject could receive social interactions consisting in either receiving on a computer screen a diagram depicting the private estimations of each member of the group (‘full information’ condition) or more simply their arithmetic mean (‘aggregated information’ condition). To test that the observed effects were due to social interactions, they also used control groups that also estimated twice but without social influence in between (‘no information’ condition). The experimental data was obtained using 144 people organized in 12 groups of 12 people. Each group was asked 6 questions, 2 in each of the three conditions.

We used our model to classify each individual by their resistance to social influence as a measure of confidence on their private information. Our proposal is then to use the geometric mean of the estimations of individuals with high social resistance as a better estimator than the WOC, as we show for the dataset from reference [9].

## Results

To understand the effect of social interactions in estimation, we first tested whether we could model each person in a group as an estimator of some quantity according to their private and the social information. We have already used this modeling approach for fish and ant groups choosing among a low number of options [14, 15]. Here we adapt it to the case of human data in which individuals estimate quantities that can take any positive real number and the distribution of estimations before social interactions is a log-normal [9, 16–18]. For the analysis of experimental data it is thus useful to take the logarithm of the raw estimations {*x*_*i*_} to obtain {*y*_*i*_ ≡ log*x*_*i*_}, whose distribution is then a Gaussian. We obtained that if before social interactions this Gaussian has mean *μ*_*p*_ and standard deviation *σ*_*p*_, $N\left(\mu_{p},\sigma_{p}\right)$, after social interactions the distribution of estimations is predicted to be of the form (see S1 Text)

$$f_{Y}\left(y\right)=N\left(w_{p}\mu_{p}+w_{s}\mu_{s},\;\sigma_{p}\sqrt{1-w_{s}}\right).$$ (1)

The predicted distribution in Eq 1 is also a Gaussian in the logarithm of estimations, but its mean and standard deviation have changed. The mean *μ*_*f*_ is at a value combination of the private mean *μ*_*p*_ and a parameter *μ*_*s*_ that summarizes the impact of social information, $\mu_{f}=w_{p}\mu_{p}+w_{s}\mu_{s}$, with $w_{p}$ and $w_{s}$ the private and social weights with values between 0 and 1 and with $w_{p}+w_{s}=1$. The form of *μ*_*s*_ in Eq 1 depends on the type of social interactions, and we considered two types. One in which each individual receives the estimations from all members of the group, for which we found that *μ*_*s*_ is of the form *μ*_*s*_ ≡ log(*x*_*s*_), where *x*_*s*_ is the geometric mean of the estimations (see S1 Text):

$$x_{s}={\left(\overset{n}{\underset{i=1}{\prod }}x_{i}\right)}^{1/n}.$$ (2)

We also considered a second form of interaction in which each individual receives only the mean of the estimations of the group, for which the social information is the mean of estimations (see S1 Text)

$$x_{s}=\frac{1}{n}\overset{n}{\underset{i=1}{\sum }}x_{i}.$$ (3)

These two types of social information impact Eq 1 differently, with only the second of them changing the mean after social interactions. This is because in the first case, as the expected value of the geometric mean of a sample following a log-normal distribution is the median of the population [19, 20], then we have on average that *x*_*s*_ = exp(*μ*_*p*_) (see S1 Text), making the mean the same as before social interactions, *μ*_*f*_ = *μ*_*p*._ In the second form of social interactions via the arithmetic mean, the expected value is $x_{s}=\text{exp}\left(\mu_{p}+\sigma_{p}^{2}/2\right)$ (see S1 Text), making the mean to shift to higher values after interactions, $\mu_{f}=\mu_{p}+w_{s}\sigma_{p}^{2}/2$. Social interactions can change not only the mean but also the standard deviation of estimations. The predicted standard deviation after social interactions in Eq 1 is reduced to $\sigma_{f}=\;\sigma_{p}\sqrt{1-w_{s}}$, more reduced the higher the social weight $w_{s}$, making the group to agree more around the final mean.

**Fig 1:**
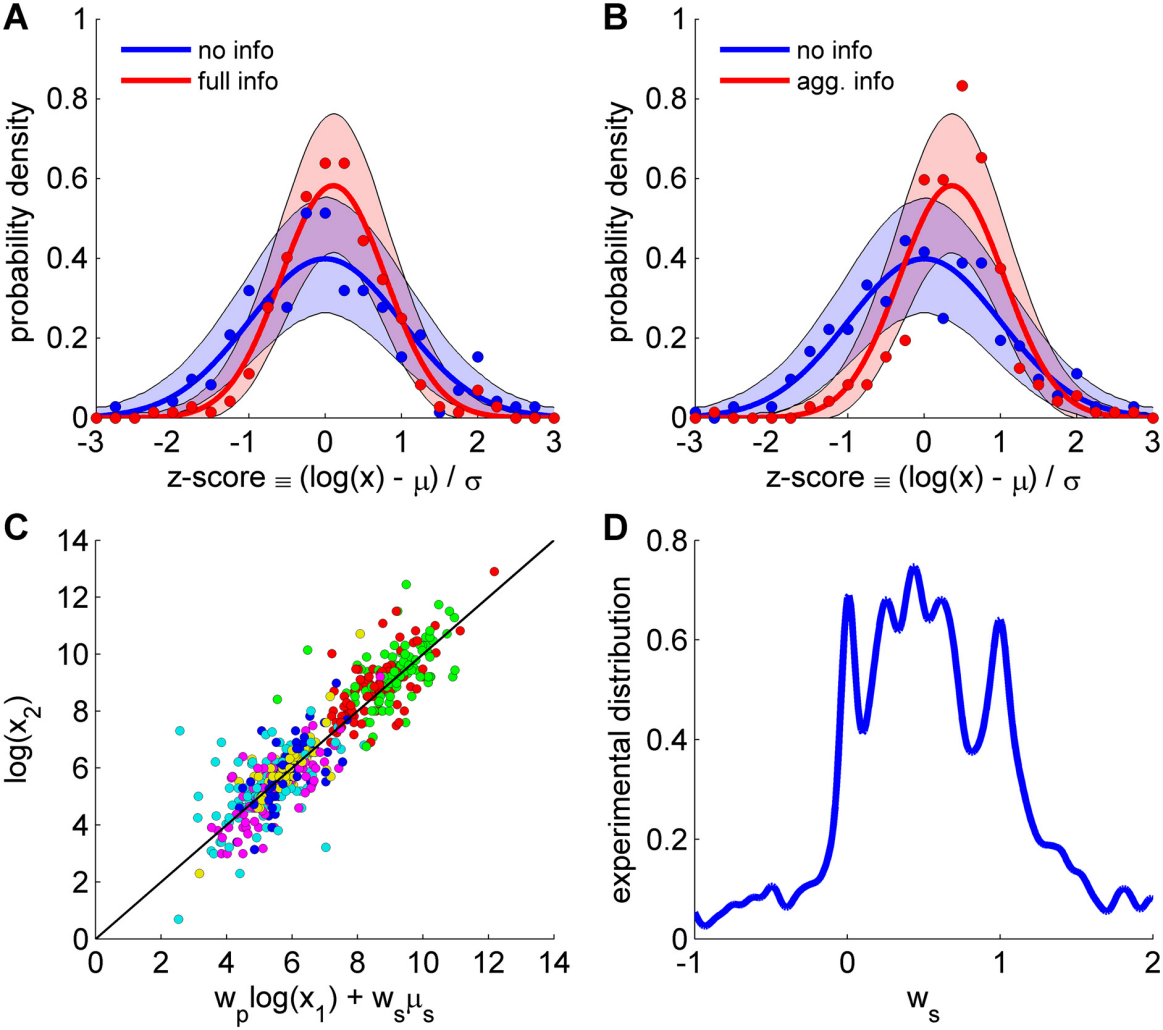
Comparison of statistical predictions against experiments of social influence. (**A**) Probability distribution of estimations before (no info, blue) and after (full info, red) receiving the estimations made by other members of the group. Estimations are pooled from 24 different experiments obtained using different groups and questions, and are plotted together using a z-score, *z* ≡ (log(*x*)-*μ*_*p*_)/*σ*_*p*_, with *x* the estimation and *μ*_*p*_ and *σ*_*p*_ the mean and standard deviation before social interactions for each experiment. Points are experimental frequencies sampled at intervals of width 0.25 and solid line is a Gaussian fit. Shadowed surface is the area in which 95 per cent of the experiments are expected by the Gaussian fit. The statistical prediction is that after social interactions the distribution of answers is also a Gaussian in the logarithmic domain with the same mean and smaller standard deviation. (**B**) Same as (A) but before (no info, blue) and after (aggregated info, red) giving subjects the mean of the estimation of all subjects. The statistical prediction is that after social interactions the distribution of answers is also a Gaussian in the logarithmic domain with higher mean and smaller standard deviation. (**C**) Real vs predicted estimations after social interactions from Eq 4 as $\text{ log}x_{2}=w_{p}\text{log}x_{1}+w_{s}\text{log}x_{s}$ using $w_{s}=0.53$. Different colors correspond to the six estimation tasks. (**D**) Distribution of experimental social weights with Gaussian kernel smoothing (see Methods). Data taken from Lorenz *et al*. [9]

We first tested that the predicted distribution in Eq 1 is consistent with the experimental data in [9]. We standardized the estimations made by each individual using a *z*-score as *z* ≡ (*y-μ*_*p*_)/*σ*_*p*_, with *y* the logarithm of the estimation and *μ*_*p*_ and *σ*_*p*_ the mean and standard deviation for each time in which a group answered a question. This transformation of variables allowed us to pool together estimations from different groups and questions, each having its own mean and standard deviation. The distribution of the *z*-score values before social influence has mean 0 and standard deviation 1, N(0,1) (Fig 1A and 1B, blue). It transforms after social influence according to Eq 1 into $N\left(0,\sqrt{1-w_{s}}\right)\;$ for the ‘full information’ condition (see S1 Text). This correctly predicts that the distribution of *z*-score values after social interactions cannot be distinguished from a Gaussian (p>0.27; Kolmogorov-Smirnov test), does not change the mean (p = 0.14, permutation test; see Methods) and reduces the standard deviation (p<10^−9^, permutation test). Unless otherwise stated, in the remainder of the paper we use permutations to obtain p-values. The predicted form $N\left(0,\sqrt{1-w_{s}}\right)\;$ gives a very good fit to the data and the standard deviation of the data corresponds to a value of the social weight in Eq 1 of $w_{s}=0.53$ (Fig 1A, red).

For the ‘aggregated information’ condition, the Gaussian distribution in Eq 1 for the *z*-score values is after social interactions of the form $N\left(w_{s}\sigma_{p}/2,\sqrt{1-w_{s}}\right)$ (see S1 Text). The value of the final mean depends on the standard deviation of estimations before the interaction, *σ*_*p*_, that is different for each of the 24 experiments [9] in which each of the 12 groups answered two questions in the ‘aggregated information’ condition. Using for each experiment the value of *σ*_*p*_ before social interactions and the value of $w_{s}$ for the same group but in the ‘full information’ condition, we can predict the shift in mean and the reduction of standard deviation for the 24 experimental cases (S1 Fig). However, a simpler analysis can be made neglecting the variability of values in *σ*_*p*_ across the 24 experiments, and instead pool all the data and consider the prediction only using the mean value $\bar{\sigma_{p}}$ as $N\left(w_{s}\bar{\sigma_{p}}/2,\sqrt{1-w_{s}}\right),$ with $\bar{\sigma_{p}}$ = 1.39, and $w_{s}=0.53$ from the ‘full information’ condition. The predicted shift in the mean, $w_{s}\bar{\sigma_{p}}/2=0.39$, and the reduction in standard deviation, $\sqrt{1-w_{s}}=0.68$, correspond well with the experimental data (Fig 1B, red) and with the more complete prediction using the sum of 24 Gaussians predicted for each experiment (S1 Fig). It correctly predicts a shift of the mean to higher values (p<10^−6^) and a reduced standard deviation (p<10^−6^) that was not found to be different to the one in the ‘full information’ condition (p = 0.45). An alternative Bayesian test [21] shows similar results for the problems studied here (see Methods and S1 Table for a summary of permutations and Bayesian significance tests). In addition, this test obtains when two quantities are likely taking the same value and not only when they are not found to be different, as in the case of the standard deviation in the ‘aggregated information’ and ‘full information’ conditions (S1 Table). In the ‘no information’ condition, subjects repeat the estimation with no social interactions in between and we found no significant change in the parameters of the distribution of estimations (S2 Fig, p>0.5; see also Bayesian test in S1 Table). This shows that the effects seen after social interactions are due to the interaction and not to a repetition of the estimation.

Once we tested the close correspondence between the statistical model in Eq 1 and the experimental data, we considered a simple model for an individual that is consistent with the statistical predictions. Specifically, an individual that privately estimates *x*_1_ and, upon reception of the social information, gives a new estimation *x*_2_ related to *x*_1_ through a linear combination in the logarithmic domain,

$$\;y_{2}=w_{p}y_{1}+w_{s}\mu_{s}.$$ (4)

with {*y*_1,2_ ≡ log*x*_1,2_}, is consistent with the statistics in Eq 1. This implies that the second estimation can be predicted from the first estimation and the social information as $\;\log x_{2}=w_{p}\text{log}x_{1}+w_{s}\text{log}x_{s}$, which is found to be a good approximation for the data with $w_{s}=0.53$ (Fig 1C). A more common rule used in the modelling of social influence in humans is the linear combination rule $x_{2}=w_{p}x_{1}+w_{s}x_{s}$ [13, 22–24], but Eq 4 is a linear combination in the logarithmic domain or, equivalently, a weighted geometric mean between private and social information,

$$x_{2}=x_{1}^{w_{p}}x_{s}^{w_{s}}.$$ (5)

So far we have assumed that each individual uses the same value of the social weight $w_{s}$. However, there might be individual differences, with some individuals less influenced by social information. Using $w_{p}+w_{s}=1$ and Eq 4 we can obtain a different value of the social weight for each individual as

$$w_{s}=\frac{y_{2}-y_{1}}{\mu_{s}-y_{1}}.$$ (6)

The distribution across the group of values of the social weight $w_{s}$ in Eq 6 shows a striking structure of individual differences (Fig 1D). Some individuals resist social influence (peak at $w_{s}=0$ in Fig 1D), others shift almost completely to the social information (peak at $w_{s}=1$), others combine private and social information (values between 0 and 1), and even some shift to values farther from the private value than the social value ($w_{s}>1$) or to values in a direction opposite to the social value ($w_{s}<0$).

**Fig 2:**
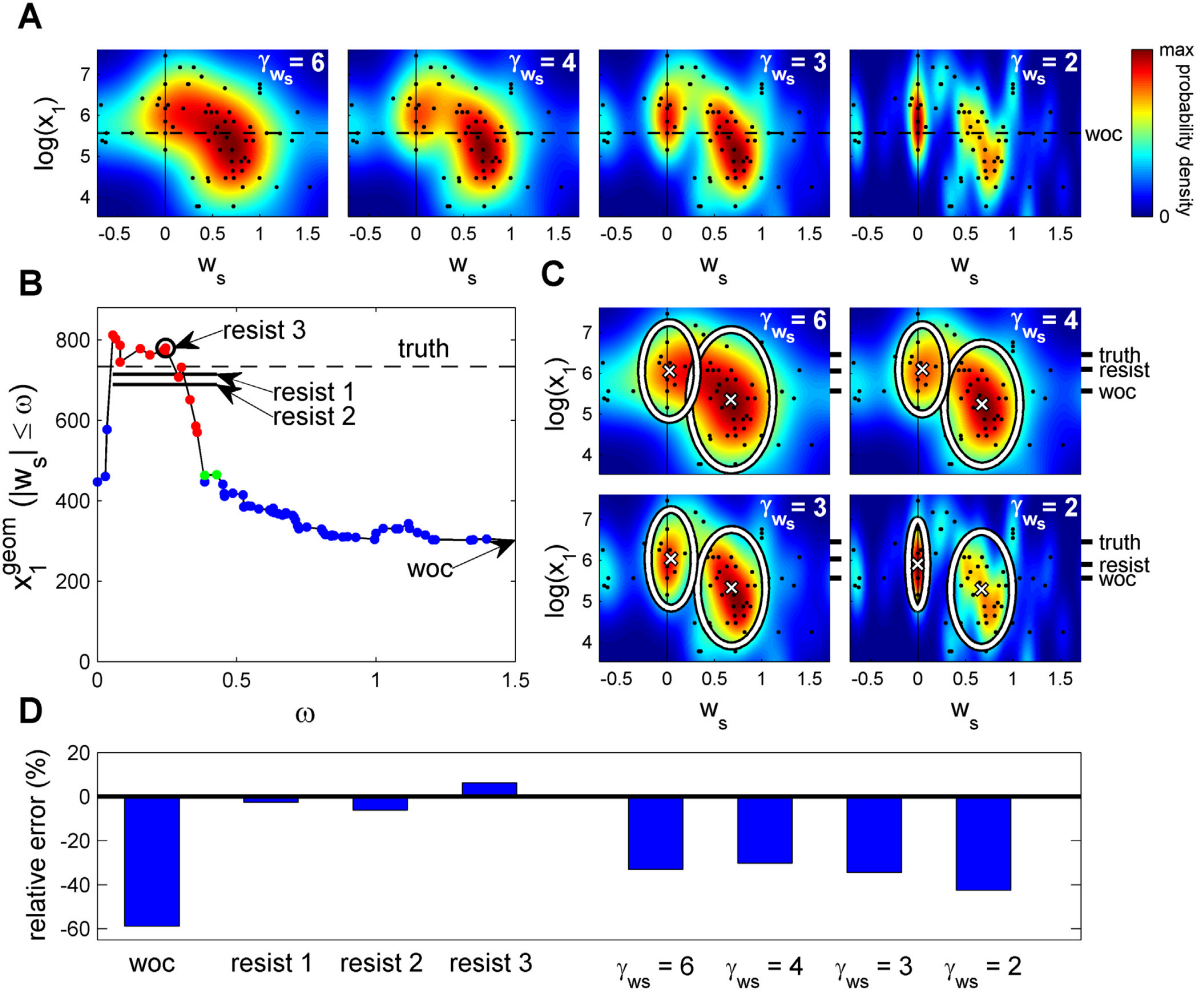
Wisdom of those resisting social influence for the question ‘What is the length of the Swiss/Italian border?’ (**A**) Joint probability density of social weights $w_{s}$ and estimations *y* = log(*x*_1_) and computed by Gaussian smoothing, Eq 7, of data (one black dot per individual). Smoothing from lowest resolution in the direction of the social weight $w_{s}$ ($\gamma_{w_{S}}$ = 6) to highest resolution ($\gamma_{w_{S}}$ = 2). (**B**) Geometric mean of estimations for groups containing individuals with social weight $\left|w_{s}\right|\leq \omega$. At low ω the groups are formed by individuals resisting social influence. Blue dots: Groups with prediction not significantly different to wisdom of the crowd (WOC). Green dots: groups significantly different from WOC at p<0.05. Red dots: p<0.01. Value labelled ‘resist 1’ computed from individuals with low social weights and contributing more the values of ω with higher significance (Eq 8). Value labelled ‘resist 2’ computed as ‘resist 1’ but not weighting the different ω differently depending on significance levels. Line labelled ‘resist 3’ corresponds to the value of ω with highest significance. (**C**) Two clusters in the space of estimations and social weights obtained using Gaussian mixtures [26]. White ellipses delimit the area that contains 95% of the probability density for each of the bivariate Gaussians [27]. (**D**) Visual summary of the relative errors made by WOC, the three variants of the method in (B) and the center of the clusters obtained at low social weight at four levels of resolution in (C). Data taken from Lorenz *et al*. [9].

We took advantage of the individuality and extracted the geometric mean of those individuals that resist social influence. To gain intuition on how to perform this extraction we considered the following exploration of the data. We obtained the joint density of social weights $w_{s}$ and private estimations *y* = log(*x*_1_) for the question ‘What is the length of the Swiss/Italian border?’ (Fig 2A). To obtain different levels of resolution, we used the following Gaussian smoothing of the data [25]

$$f\left(w_{s},y\right)=\frac{1}{2\pi \sigma_{w_{s}}\sigma_{y}n}\sum_{i=1}^{n}\text{exp}\left(-\frac{{\left(w_{s}-w_{s,i}\right)}^{2}}{2\sigma_{w_{s}}^{2}}-\frac{{\left(y-y_{i}\right)}^{2}}{2\sigma_{y}^{2}}\right)$$ (7)

with $w_{s,i}$ and *y*_*i*_ = log(*x*_1,*i*_) the social weight and the private estimation of individual *i*, respectively, $\sigma_{y}\equiv {\hat{\sigma }}_{y}n^{-1/\gamma_{y}}$ and $\sigma_{w_{s}}\equiv {\hat{\sigma }}_{w_{s}}n^{-1/\gamma_{w_{s}}}$ with ${\hat{\sigma }}_{y}$ and ${\hat{\sigma }}_{w_{s}}$ the sample standard deviation of each variable. We varied the resolution coefficient $\gamma_{w_{s}}$ while keeping *γ*_*y*_ at its optimal value of *γ*_*y*_ = 6 [25] to see whether there is a consistent tendency for individuals with different social weights to give different estimations (Fig 2A). At the lowest resolution considered, $\gamma_{w_{s}}=6$, there is a clear tendency of individuals with lower social weight to give higher estimations of the border length between Switzerland and Italy (Fig 2A, $\gamma_{w_{s}}=6$). At resolution $\gamma_{w_{s}}=$ 2, 3 and 4 the density splits into two peaks, one at high $w_{s}$ and another at low $w_{s}$ (Fig 2A, $\gamma_{w_{s}}=$ 2,3,4). It is thus clear that for this question the individuals with lower social weight tend to give higher estimations.

We then extracted the individuals with lowest social weight. A simple method consists in extracting all individuals with a social weight below the value that gives a result significantly different to WOC (Fig 2B). Specifically, we started from the complete group and its geometric mean as the WOC value. For this case, the WOC value is 302 km (Fig 2B). We then eliminated individuals one by one from highest to lowest values of the social weight keeping those with $\left|w_{s}\right|\leq \omega$, with ω a decreasing positive real number. With the remaining individuals, we computed the geometric mean. For ω in the interval between 0.1 and 0.5 of individuals with high resistance, the geometric mean increases to values close to 800 km. At the lowest values of ω there is a drop in the geometric mean, but the number of individuals is also low. To isolate the relevant individuals, we found which values of ω give a geometric mean significantly different from the WOC (Fig 2B, green dots for p<0.05 and red dots for p<0.01). The significant values of ω are in the interval from 0.06 to 0.45, which correspond to groups whose geometric mean lies between 816 and 464 km, respectively. We then tested that we obtain similar estimations using the complete interval of significant values of ω or only the value of ω giving the highest significance. Specifically, for the complete interval of significant ω we used the following measure that weighted more the values of ω with higher significance as

$$\text{resist 1}\equiv \frac{\int_{0.5}^{0}q\left(\omega \right)x_{1}^{geom}\left(\left|w_{s}\right|\leq \omega \right)d\omega }{\int_{0.5}^{0}q\left(\omega \right)d\omega }$$ (8)

with $x_{1}^{geom}\left(\left|w_{s}\right|\leq \omega \right)$ the geometric mean of the estimations of individuals with social weight $\left|w_{s}\right|\leq \omega$, q(ω)=0.05−p(ω)  if the p-value obeys p(ω)<0.05  and q(ω)=0 otherwise, and only counting those groups with sufficiently low social weight, ω≤0.5. The prediction obtained in this way is 714 km, that deviates only -2.7% from the true value of 734 km while the WOC value of 302 km deviates -59% (Fig 2B, ‘resist 1’, ‘truth’ and ‘WOC’). An alternative to Eq 8 would also use the values of ω giving significance but weighted all of them equally, giving 689 km, -6.2% off the true value (Fig 2B, ‘resist 2’). Another variant would only take into account a single value of ω with the highest significance (p = 0.0002) that corresponds to ω=0.25. This gives the prediction of 780 km, 6.3% off the true value (Fig 2B, ‘resist 3’). The three variants give very similar predictions and a large improvement over WOC.

We also used a second class of methods based on the finding that resisting individuals can form peaks in the joint distribution of estimations and social weight (Fig 2A). Methods using the peaks will in general use less individuals but should be valuable when the peaks are clear in the distribution, that is, when they are sharp and separated from other peaks. Specifically, we used clustering by Gaussian mixtures [26]. The advantage of this method is that, although it depends on the distribution and therefore on the value of the resolution $\gamma_{w_{s}}$, it is very robust to changes in its value. For the question about the length of the Swiss/Italian border, we obtained that the geometric mean of the cluster of people with low social weight is 422, 481, 512 and 491 km for $\gamma_{w_{s}}$ = 2, 3, 4 and 6, respectively (Fig 2C). In particular, it is not necessary that the value $\gamma_{w_{s}}$ chosen for the clustering corresponds with a distribution showing peaks. For example, the distribution with $\gamma_{w_{s}}$ = 6 does not show peaks and it is clustered into approximately the same two clusters than the distribution with $\gamma_{w_{s}}$ = 3 that shows two clear peaks. The values obtained are -42%, -34%, -30% and -33% off the true value of 734 km. The cluster at high social weight correspond to individuals with larger errors (-69%, -67%, -71% and -67% for $\gamma_{w_{s}}$ = 2, 3, 4 and 6, respectively). WOC is typically a value between the ones at low and at high social weights, here 302, -59% off the true value.

**Fig 3:**
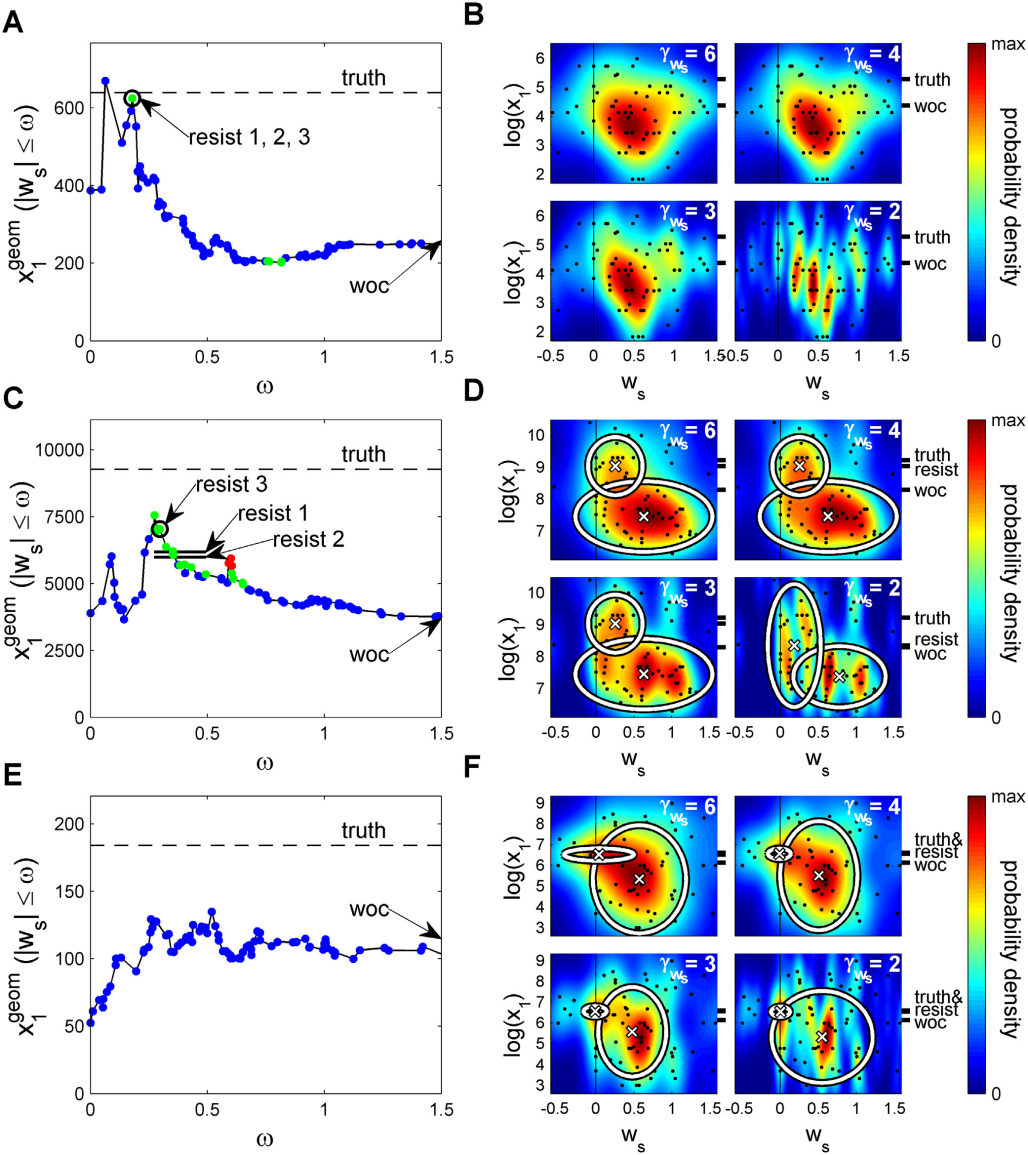
Wisdom of those resisting social influence for three questions. Analysis as in Fig 2B and 2C but for the questions (**A, B**) ‘How many rapes were officially registered in Switzerland in 2006?’, (**C, D**) ‘How many assaults were officially registered in Switzerland in 2006?’, and (**E, F**) ‘What is the population density of Switzerland in inhabitants per square kilometer?’ See S3 Fig for densities in (**D**) and (**F**) without ellipses. Data taken from Lorenz *et al*. [9].

So far we have seen that using the individuals with lowest social weight we can estimate ‘What is the Swiss/Italian border length?’ better than using WOC. The results were robust under changes in the method to extract the individuals with low social weights, with a total of 7 variants of the methods used improving over WOC (Fig 2D). We then applied the same methods to the remaining 5 questions from the experiments in [9]. We found a subpopulation with a significant resistance to social influence in 3 of the remaining questions (Fig 3 and Table 1 for a summary; see S4 Fig for the other two questions).

**Table 1:** Comparison of true value, ‘wisdom of the crowds’ (WOC) and the prediction from the subgroup of individuals resisting social information. **resist 1** computed from individuals with low social weights and contributing more the values of ω with higher significance (Eq 8). **resist 2** computed as ‘resist 1’ but not weighting the different ω differently depending on significance levels. **resist 3** corresponds to the value of ω with highest significance. $\gamma_{w_{s}}$ = 6, 4, 3, 2 give the central values of the peaks at low social weights obtained from a Gaussian mixture at a resolution in the direction of social weight $w_{s}$ obtained introducing the values of $\gamma_{w_{s}}$ in Eq 7. **Border**, ‘What is length of the Swiss/Italian border?’ **Rapes**, ‘How many rapes were officially registered in Switzerland in 2006?’ **Assaults**, ‘How many assaults were officially registered in Switzerland in 2006?’ **Population**, ‘What is the population density of Switzerland in inhabitants per square kilometer?’

Question	truth	WOC	resist 1	resist 2	resist 3	$\gamma_{w_{s}}=6$	$\gamma_{w_{s}}=4$	$\gamma_{w_{s}}=3$	$\gamma_{w_{s}}=2$
Border	734	302	714	689	780	491	512	481	422
		(-59%)	(-2.7%)	(-6.2%)	(+6.3%)	(-33%)	(-30%)	(-34%)	(-42%)
Rapes	639	257	624	624	624	-	-	-	-
		(-60%)	(-2.3%)	(-2.3%)	(-2.3%)				
Assaults	9272	3685	6170	5984	7037	7699	7699	7699	3881
		(-60%)	(-33%)	(-35%)	(-24%)	(-17%)	(-17%)	(-17%)	(-58%)
Population	184	115	-	-	-	171	177	177	174
		(-38%)				(-7.3%)	(-4.0%)	(-4.0%)	(-5.7%)

For the question of ‘*Number of rapes in 2006 in Switzerland*’ the geometric mean of individuals of low social weight as measured by Eq 8 and its two variants gives the same value as there is a single significative group at a value of 624, much larger than the WOC result of 257 (Fig 3A, ‘resist 1,2,3’). This corresponds to a much smaller error (-2.3%) than the WOC (-60%) respect to the truth at 639. The distribution of estimations does not show a structure of two peaks separated at low and high social weight (Fig 3B, $\gamma_{w_{s}}$ = 3,4,6) and at high resolution there are too many peaks with very few individuals each (Fig 3B, $\gamma_{w_{s}}$ = 2) so a method based on peaks is not appropriate for this question.

For the ‘*Number of assaults in 2006 in Switzerland*’, the geometric mean in Eq 8 and the two variants considered have a large deviation from the WOC value of 3685 to 6654, 6313 and 7557, respectively (Fig 3C, ‘resist1’,’resist 2’,’resist 3’). They correspond to errors of -28%, -32% and -18%, respectively, much lower than the -60% error of WOC. The clustering method obtains the same value of 7699 for $\gamma_{w_{s}}$ = 3, 4 and 6 (Fig 3D, $\gamma_{w_{s}}$ = 3,4,6) and for $\gamma_{w_{s}}$ = 2 the resolution is too high and reveals at least four peaks with very few individuals per peak (Fig 3D, $\gamma_{w_{s}}$ = 2). For $\gamma_{w_{s}}$ = 3,4, and 6 the error is -17% of the true value 9272 compared to the -60% error of the WOC of 3685.

For the question about the ‘*Population density of Switzerland*’ the geometric mean in Eq 8 does not find a subpopulation resisting social influence with estimations significantly different to WOC (Fig 3E). The clustering method finds for $\gamma_{w_{s}}$ = 2,3,4 and 6 the values 174, 177, 177 and 171, respectively (Fig 3F, $\gamma_{w_{s}}$ = 2,3,4,6). Compared to the true value of 184, these values are -5.7%, -4.0%, -4.0% and -7.2% off the true value of 184 while the WOC value of 115 is -38% off.

Our analysis shows that estimation is improved when there is a subpopulation significantly resisting social influence. The seven variants of the methods improve upon WOC and in many cases the improvement is very large (Table 1). The success of the method rests in the correlation between resistance to social influence and closeness to the true value seen in the data. It is also interesting to consider some properties of the resisting individuals. The proportion of these individuals is 25±13% using the methods based on Eq 8 and 10±3% for the methods based on the peaks of the distribution. The individuals that resist social influence are not the same in all questions. We only find a significant overlap between questions 1 and 2 (S5A Fig, p<0.05).

Resistance to social information may be viewed as a behavioral measure of confidence, and the estimation of those resisting social influence as ‘wisdom of the confident’. Its success is not a trivial result as other measures of confidence like declared confidence in a scale from 1 to 6 does not improve accuracy [28–31]. We thus decided to compare why the two measures give different results. We found a significant but very low correlation between resistance to social information and declared confidence (S5B Fig, p<0.001, R^2^ = 0.03). While there are approximately equal numbers of resisting and non-resisting individuals (Fig 1D), most of the population declares low values of confidence, even the majority of those resisting social influence (S5C Fig, triangles). Individuals declaring high values of confidence (S5D Fig, triangles), in general resist social influence more than those with low values, but a relevant proportion does not resist social influence. The two measures are correlated but are very different and it is then unsurprising than a method like the one proposed here for social resistance does not work for declared confidence (S6 Fig).

## Discussion

We have here proposed to extract information from the collective using those individuals resisting social influence. The methods proposed extract the information a collective considers of high private quality. We obtained better collective estimations than the ‘wisdom of crowds’ [1–9] using the data from [9], especially for cases in which the crowd shows a very large bias. The methods work because resistance to social influence correlates with closeness to the true value. The correlation does not need to be very strong, that is, we do not need experts [10–12]. Instead, we use the geometric mean of those individuals that get influenced less by social information and this group can still show a large standard deviation.

We used two types of methods. One based on Eq 8, taking all individuals below a value of social weight that give a result different from WOC. This method gave predictions very close to true values for those cases in which the joint distribution of estimations and social weight does not show a complex structure at low social weights. When this method does not give significant results, one can resort to a method based on clustering in the space defined by estimations and social weights. This second type of methods takes into account less individuals, but we found they improve upon WOC. The two methods together can be used to understand the relevant subjects in the estimation. For example, Eq 8 does not give significant results for the question on the ‘*Population density of Switzerland*’ (Fig 3E). Inspection of the density shows that while there is a strong peak at low social weight with an estimation very different from WOC (Fig 3F), there are individuals giving much lower estimations and thus making the geometric mean of individuals with low social weight not different from WOC.

Our proposal makes use of individuality to improve upon WOC. It is interesting to speculate what type of individuality is most compatible with our results. One type of individuality would simply be that all individuals use a similar procedure to answer a question but their levels of noise are different. One way to model this would be to extend our models to incorporate that all individuals are most likely to give the correct answer but they have different levels of noise (S1 Text). This model gives very poor predictions (S1 Text). The reason is that the data seems more compatible with different subgroups of people with different biases from the truth, for example the low and high peaks in the joint density in Fig 2A. This can be modelled in that the most probable estimation is shifted away from the true value with different biases in different individuals. As biases are defined respect to truth, this extension of the models would not be predictive. Instead, we propose the methods in the main text, by which we extract the subgroup of individuals of low social weight as the more accurate ones on average.

The idea that different individuals or subgroups of individuals have different biases is compatible with the existence in the population of different procedures to solve a problem, each of them with a different bias. According to this view, a possible origin of the data for the question about the Swiss/Italian border as an example could be the following. This question might be answered estimating the approximate length of a straight line separating the two countries, which is 288 km as measured from a map in http://www.freemaptools.com/measure-distance.htm. Interestingly, the cluster of individuals with highest social weight is characterized by an estimation of 216±157 km compatible with these very low values. A procedure more sophisticated than simply the length of a straight line consists in using the shape of the border. Another procedure is to use memorized data to retrieve its value. The cluster at low social weight is characterized by an estimation of 512 ±269 km and the geometric mean at low social weights by values in the interval 650–800 km, compatible with these more sophisticated procedures. This idea of different procedures might also explain the different susceptibilities to social information. Those individuals using the shape of the Swiss/Italian border would in general not consider as very important social information with values so much lower than their estimations. This is because these values would be incompatible with the shape, for example values closer to a straight line. In contrast, individuals using a straight line approach might be willing to consider higher values, as they might have only taken this approach as a very rough approximation they could make because they had difficulties finding how to estimate the full shape. All individuals might declare low confidence levels as they can be very noisy within their approach, but they might still consider differently values more compatible with other approaches.

A second and complementary explanation of individuality is that individuals have different levels of expertise on the subject or even in general exercises of estimation. This level of expertise is probably not high enough for the individuals to declare it, but it would be enough to act upon it when confronted with social influence.

The methods proposed to improve upon WOC do not correspond to a common situation in which humans interact naturally. Instead, it is a protocol that can be used to extract high quality information in human collectives even if it is present only in a minority of the group. Its value relies on improving upon WOC by eliminating the people that are not confident in their private estimations. And using how much each individual is influenced by others as a measure of confidence seems to extract the correct individuals, unlike methods based on declared confidence [28–31]. Our results point to measures of confidence not based on declaration as a means to gather high quality private information in a group. Response time, perseverance or pay-offs in decision systems might be implementations to test experimentally. An open problem is in which circumstances social influence or these other measures of confidence can be used by humans to improve individual and collective decisions in naturalistic settings.

## Methods

Experimental data from Lorenz et *al*. [9] can be downloaded from http://www.pnas.org/content/108/22/9020?tab=ds. In those experiments subjects were asked to estimate five consecutive times for each of six questions described in the main text.

### Smoothing of distributions

The distributions were calculated using Gaussian kernel smoothing [25]. The 1D version of Gaussian kernel smoothing was applied for social weights $w_{s}$ in Fig 1D.

$$f\left(w_{s}\right)=\frac{1}{\sqrt{2\pi }\sigma n}\overset{n}{\underset{i=1}{\sum }}e^{-\frac{{\left(w_{s}-w_{s,i}\right)}^{2}}{2\sigma^{2}}},$$ (9)

with $\;\left\{w_{s,i}\right\}$ the values of the social weights obtained from experiments using Eq 6, n the length of the sample and $\sigma \equiv \hat{\sigma }n^{-1/\gamma }$ the bandwidth with $\hat{\sigma }$ the standard deviation of the sample and *γ* the resolution coefficient. We set the resolution coefficient to half its optimal value [25], $\gamma =\frac{5}{2}$, a value that allows the visualization of the main structure of the distribution. We were interested in the interval [0,1] and did not then consider points outside (-1,2) in our calculations of the bandwidth, avoiding tail effects. The 2D case of Gaussian kernel smoothing is described in the main text, Eq 7.

### Significance tests used for the difference of means or variances

A complete list of significance tests can be found in S1 Table. In the main text, unless otherwise stated, we computed p-values explicitly without assumptions about the data as the probability that the experimental result is obtained at random. For example, to find whether two distributions have a significantly different value of some parameter *θ* (in our case, the mean or the variance), we performed a permutations method. We mixed the two samples and randomly divided the resulting set into two subsets. Then, we computed the sample value of the parameter in each of the subsets and extracted the difference *d* ≡ |*θ*_1_-*θ*_2_|. We repeated this process 10^6^ times, obtaining a distribution of differences *d*. The significance p is the proportion of *d* values bigger than the difference of the parameters between the two original samples.

To find whether the group of individuals with $w_{s}\leq \omega$ in Figs 2 and 3 has geometric mean significantly different from WOC, we used the following procedure. Each ω corresponds to a subgroup of $n_{\omega }$ individuals. We obtained 10^5^ random sets of $n_{\omega }$ estimations from the whole crowd and computed the geometric mean of each set, *g*. The significance of $x_{1}^{geom}\left(w_{s}\leq \omega \right)$ is the proportion of values of *g* at least as far to the wisdom of the crowd (geometric mean) as $x_{1}^{geom}\left(w_{s}\leq \omega \right)$.

### Significance test used for the method using the distributions

To divide the region of maximum density into two clusters, we performed an Expectation Maximization (EM) algorithm to obtain a mixture of two Gaussians [26]. More specifically, for each value of $\gamma_{w_{s}}$ we selected those individuals whose social weight and estimation $\left(w_{si},\log x_{i}\right)$ lied in the zone of maximum probability, defined as that where the probability in Eq 7 is at least equal than half of the maximum. Then an EM algorithm was applied to the selected data points to find the maximum likelihood estimates of the parameters of a Gaussian mixture with two components.

### Significance test of whether two questions share the same resisting individuals

To find whether two questions shared a significant number of individuals with low $\left|w_{s}\right|$, we used the exact expression for the probability that two samples from a finite population have a certain number of elements in common (see S1 Text).

## Supporting Information

S1 Fig Distribution of estimations before and after receiving the mean estimation for each experiment. Same analysis as in Fig 1B, but for each of the 24 experiments (**A**) and the sum of the 24 Gaussians (**B**) before (blue) and after (red) receiving the mean value of the estimations. Points are experimental frequencies at intervals of width 1 (**A**) and 0.25 (**B**). Shadowed surface is the area where the 95 per cent experiments are expected given the theoretical fit. Data taken from Lorenz *et al*. [9]
(TIFF)

S2 Fig Distributions of estimations without interactions. As Fig 1A and 1B in main text, probability distribution of z-score estimating twice without interactions in between (first: blue, second: red). Points are experimental frequencies at intervals of width 0.25. Solid line is a Gaussian fit. Shadowed surface is the area where the 95 per cent experiments are expected given the theoretical fit. Data taken from Lorenz *et al*. [9]
(TIFF)

S3 Fig Joint probability distributions without ellipses. (**A**) ‘How many assaults were officially registered in Switzerland in 2006?’, and (**B**) ‘What is the population density of Switzerland in inhabitants per square kilometer?’ Data taken from Lorenz *et al*. [9]
(TIFF)

S4 Fig Collective estimations of those resisting social influence for two questions not analyzed in main text. Same as in Fig 3 of main text but for the two remaining experimental questions: (**A, B**) ‘How many murders were officially registered in Switzerland in 2006?’, and’ (**C, D**) ‘How many more inhabitants did Zurich gain in 2006? No significant subgroup is found using the method of the geometric mean value (**A, C**). Using the joint distribution (**B, D**), we do not find a clear separation into a peak for a group of individuals resisting social influence (*w*_*s*_<0.5)and a peak for individuals not resisting the influence (*w*_*s*_>0.5) Data taken from Lorenz *et al*. [9]
(TIFF)

S5 Fig Characterization of individuals resisting social information. (**A**) Significance of the coincidence of resisting individuals (*w*_*s*_<0.5) for every pair of the 4 questions analyzed in main text. There is only a significant overlap of individuals resisting influence for questions 1 and 2 (‘What is the length of the border between Switzerland and Italy in kilometers?’ and ‘How many rapes were officially registered in Switzerland in 2006?’). (**B**) Correlation of social weight (only for |w_s_|≤1) and declared confidence is significant (p<0.0003) but weak (R^2^ = 0.03) respect to linear regression (straight line). Triangles at mean social weight for each confidence value. In colors the joint distribution of social weights and confidence values, showing large dispersion from regression line. (**C**) Probability of the declaration of confidence for individuals resisting (red triangles) and not resisting (blue circles) social influence. (**D**) Probability that an individual has a social weight when they declare a low (blue circles) and high confidence (red triangles). Data taken from Lorenz *et al*. [9]
(TIFF)

S6 FigCollective estimations for individuals declaring confidence.We used a method analogous to that of Figs 2B, 3A, 3C and 3E in main text but for declared confidence instead of social weight. Geometric mean of individuals declaring a value of confidence (*conf*) in their estimation higher or equal than an integer κ. No value is found to be significant (p_min_>0.08, $\bar{\text{p}}>0.54$). The experimental questions are: (**A**) ‘What is the length of the Swiss/Italian border?’, (**B**) ‘How many rapes were officially registered in Switzerland in 2006?’, (**C**) ‘How many assaults were officially registered in Switzerland in 2006?’, (**D**) ‘What is the population density of Switzerland in inhabitants per square kilometer?’, (**E**) ‘How many murders were officially registered in Switzerland in 2006?’, and (**F**) ‘How many more inhabitants did Zurich gain in 2006?’ Data taken from Lorenz *et al*. [9](TIFF)

S1 Text Supplementary Text. (DOCX)

S1 Table Kolmogorov-Smirnov, permutations and Bayesian significance tests. Summary of the results of the significance tests in main text. **Kolmogorov-Smirnov** tests were run with Matlab to check normality. **Permutations** method were performed as explained in the main text (Methods) to test for the equality of means and equality of variances. For the no difference of means, two sample t-tests were run with Matlab to check compatibility with permutations method. For the no difference of variances, two sample F-tests were run with Matlab with the same purpose. No discrepancies in the acceptance/rejection of the null hypothesis were found in any of the no difference tests. **Bayesian tests** are based on the likelihood of the experimental data given a certain value of the parameters. More specifically, we follow the reference [21] in the main text: Kruschke JK (2013) Bayesian estimation supersedes the t test. J. Exp. Psychol. Gen. 142(2), 573. The method generates a probability distribution of the most credible values of the parameters (or their difference for two distribution comparison). If a value falls outside the 95% highest density interval (HDI) then it is not considered to be a credible value of the parameter or difference of parameters. For the distribution to be considered credibly normal, a value for the degrees of freedom parameter of log_10_(*v*) > log_10_(30)*≈*1.48 is required. Only one discrepancy was found with the null hypothesis methods, and the Bayesian test cannot accept the normality of the estimation distribution generated in the second trial of the ‘aggregated information’ condition. Although the Kolmogorov-Smirnov test did not reject the normality hypothesis, the p-value was slightly above 0.05. In the main text and in S1 Fig this poor value is explained by the fact that the distribution is better explained the sum of 24 Gaussians with very similar parameters.
(DOCX)
