# Correction: Improving Collective Estimations Using Resistance to Social Influence

**DOI:** 10.1371/journal.pcbi.1004713

**Published:** 2016-01-06

**Authors:** The PLOS Computational Biology Staff

The funding statement for this article should read as follows:

“We acknowledge funding from Spanish Ministerio de Economía y Competitividad (BFU2009-09967 and BFU2013-49512-EXP) to G.G.d.P including a doctoral contract to G.M. and from the Champalimaud Neuroscience Programme (Portugal). The funders had no role in study design, data collection and analysis, decision to publish, or preparation of the manuscript.”
