# Supplementary figures and images for: Improving Collective Estimations Using Resistance to Social Influence
Source: PLoS Comput Biol. 2015 Nov 13;11(11):e1004594. doi: 10.1371/journal.pcbi.1004594 (PMC4643903; doi:10.1371/journal.pcbi.1004594)

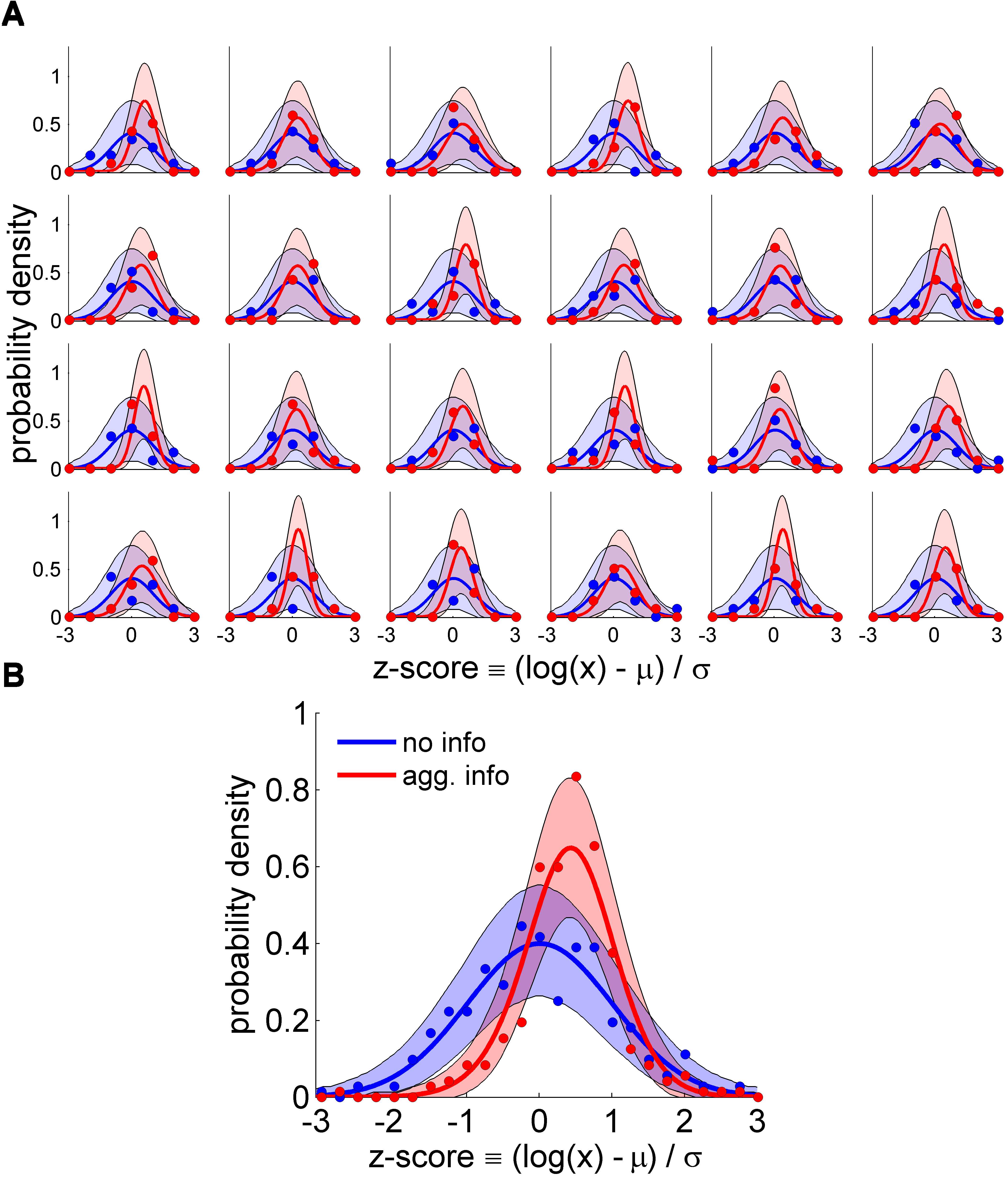

Supplement: S1 Fig — Same analysis as in Fig 1B, but for each of the 24 experiments (A) and the sum of the 24 Gaussians (B) before (blue) and after (red) receiving the mean value of the estimations. Points are experimental frequencies at intervals of width 1 (A) and 0.25 (B). Shadowed surface is the area where the 95 per cent experiments are expected given the theoretical fit. Data taken from Lorenz et al. [9] (TIFF) [file pcbi.1004594.s001.tiff]

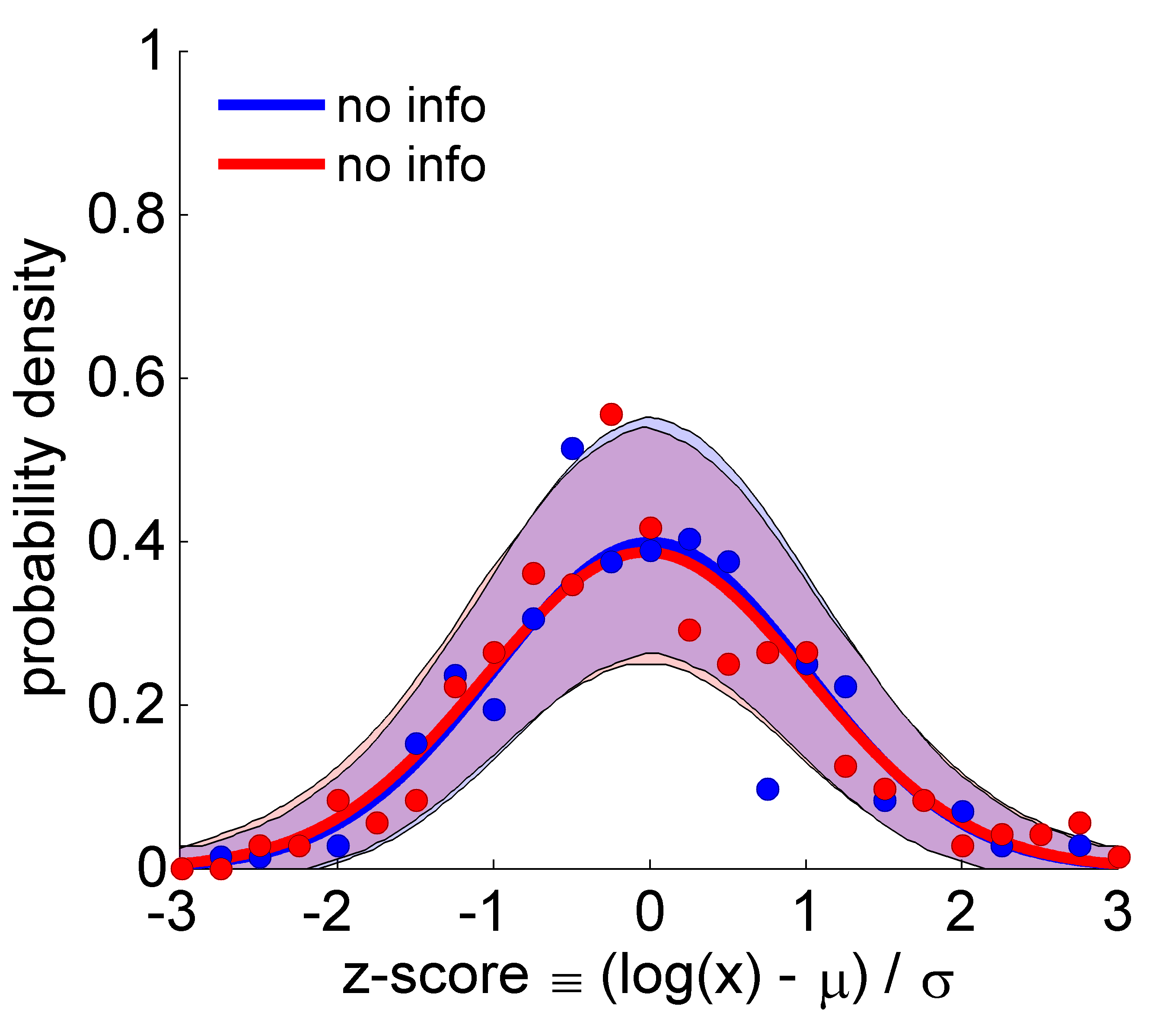

Supplement: S2 Fig — As Fig 1A and 1B in main text, probability distribution of z-score estimating twice without interactions in between (first: blue, second: red). Points are experimental frequencies at intervals of width 0.25. Solid line is a Gaussian fit. Shadowed surface is the area where the 95 per cent experiments are expected given the theoretical fit. Data taken from Lorenz et al. [9] (TIFF) [file pcbi.1004594.s002.tiff]

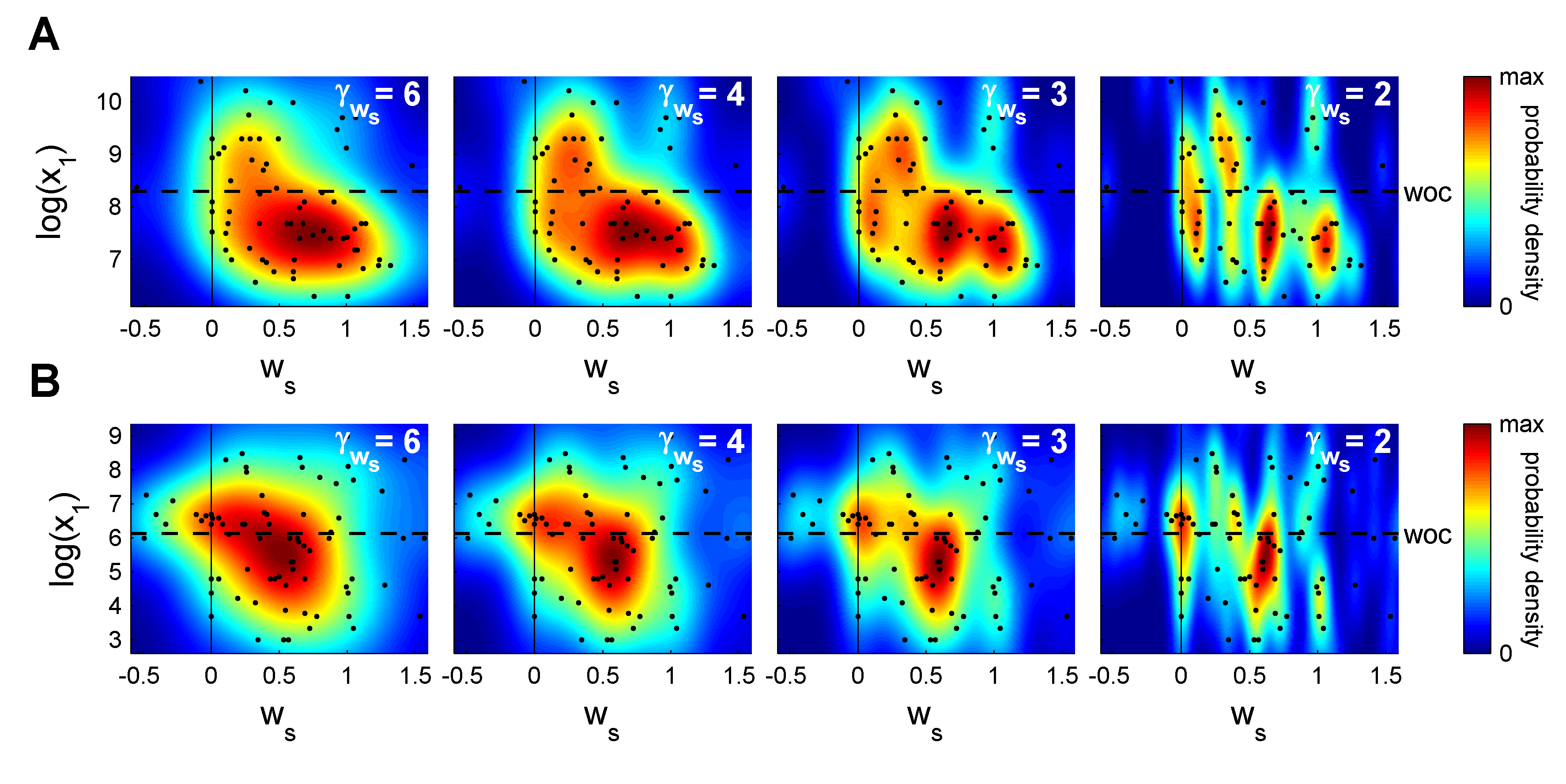

Supplement: S3 Fig — (A) ‘How many assaults were officially registered in Switzerland in 2006?’, and (B) ‘What is the population density of Switzerland in inhabitants per square kilometer?’ Data taken from Lorenz et al. [9] (TIFF) [file pcbi.1004594.s003.tiff]

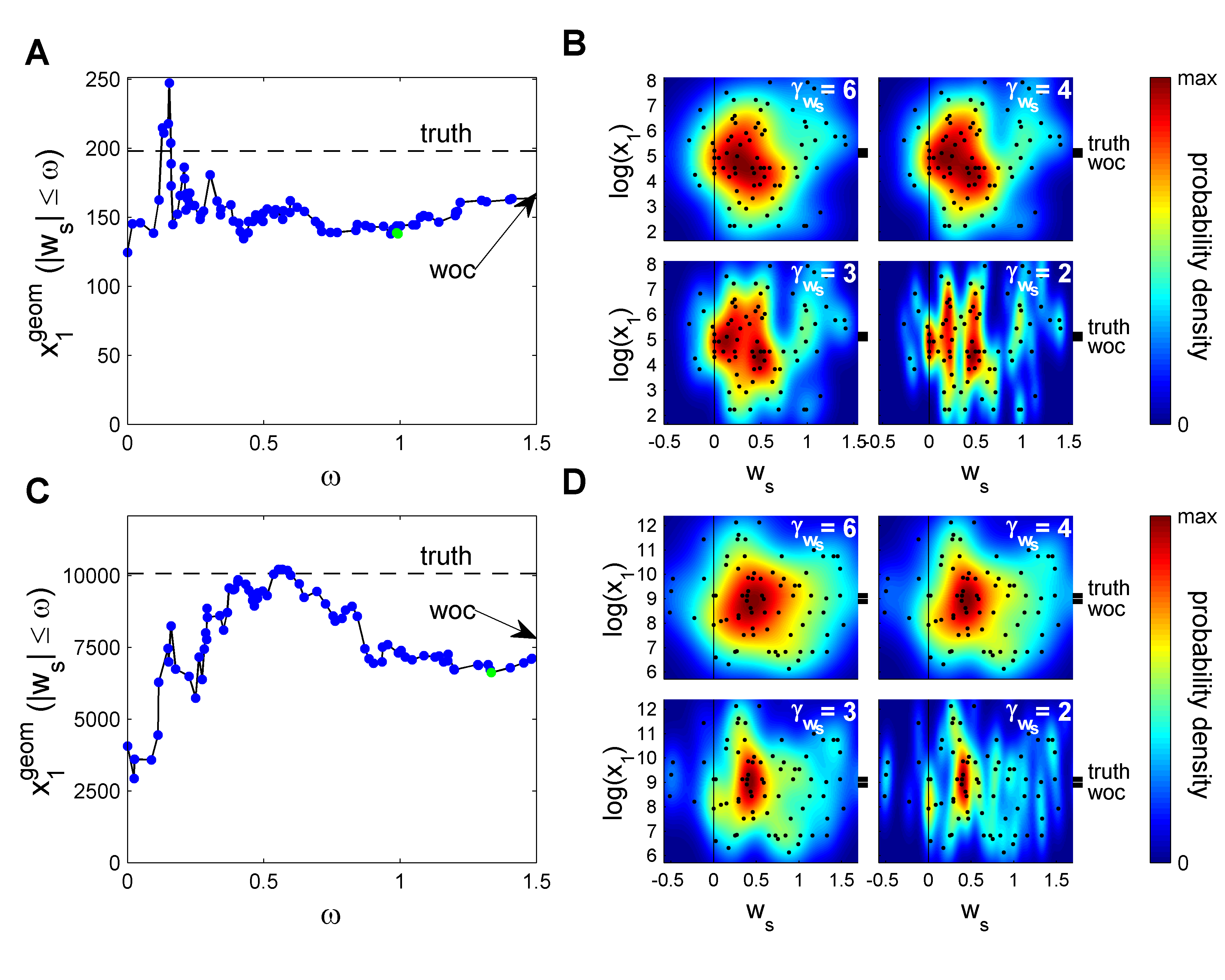

Supplement: S4 Fig — Same as in Fig 3 of main text but for the two remaining experimental questions: (A, B) ‘How many murders were officially registered in Switzerland in 2006?’, and’ (C, D) ‘How many more inhabitants did Zurich gain in 2006? No significant subgroup is found using the method of the geometric mean value (A, C). Using the joint distribution (B, D), we do not find a clear separation into a peak for a group of individuals resisting social influence (w s<0.5)and a peak for individuals not resisting the influence (w s>0.5) Data taken from Lorenz et al. [9] (TIFF) [file pcbi.1004594.s004.tiff]

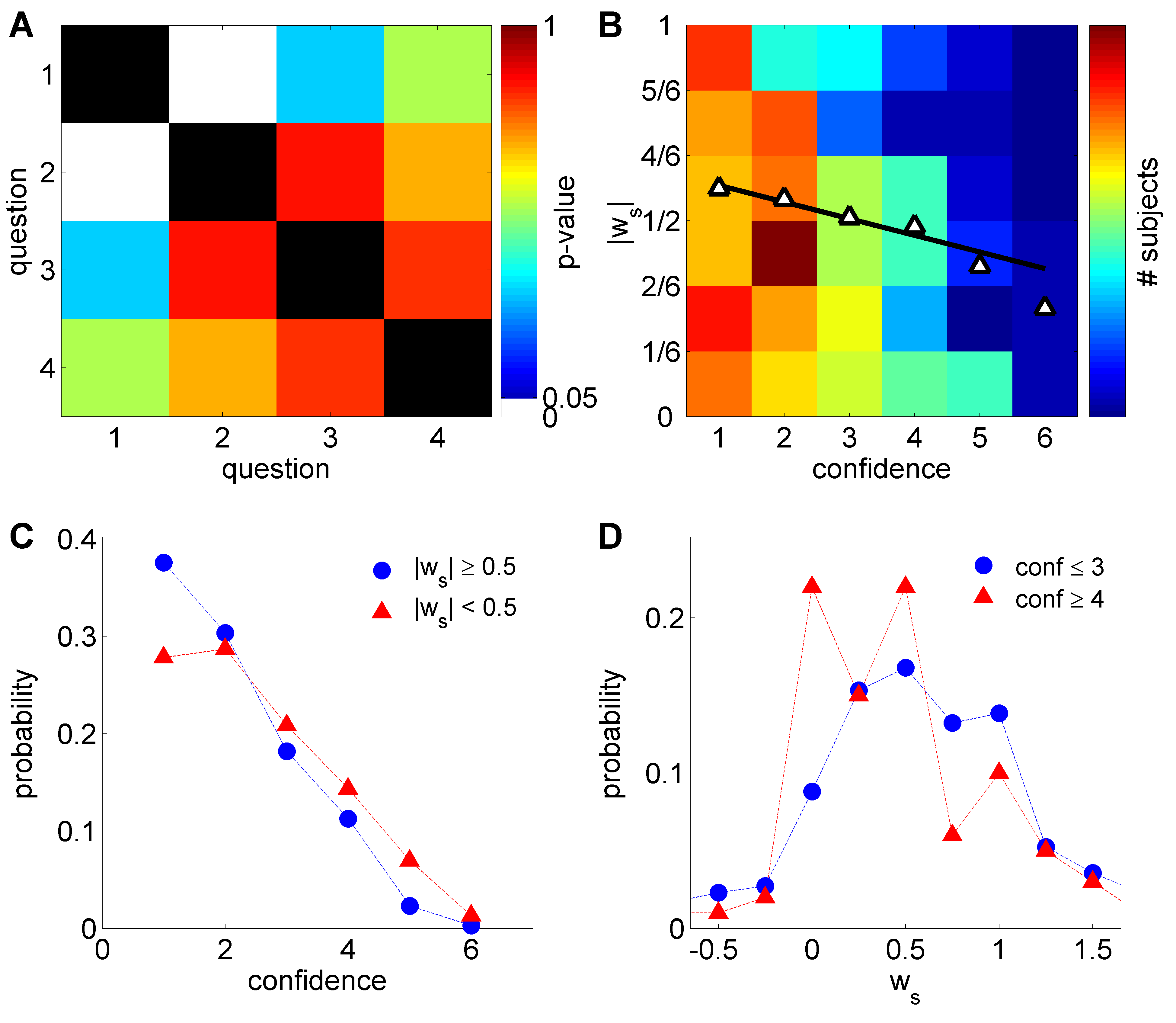

Supplement: S5 Fig — (A) Significance of the coincidence of resisting individuals (w s<0.5) for every pair of the 4 questions analyzed in main text. There is only a significant overlap of individuals resisting influence for questions 1 and 2 (‘What is the length of the border between Switzerland and Italy in kilometers?’ and ‘How many rapes were officially registered in Switzerland in 2006?’). (B) Correlation of social weight (only for |ws|≤1) and declared confidence is significant (p<0.0003) but weak (R2 = 0.03) respect to linear regression (straight line). Triangles at mean social weight for each confidence value. In colors the joint distribution of social weights and confidence values, showing large dispersion from regression line. (C) Probability of the declaration of confidence for individuals resisting (red triangles) and not resisting (blue circles) social influence. (D) Probability that an individual has a social weight when they declare a low (blue circles) and high confidence (red triangles). Data taken from Lorenz et al. [9] (TIFF) [file pcbi.1004594.s005.tiff]

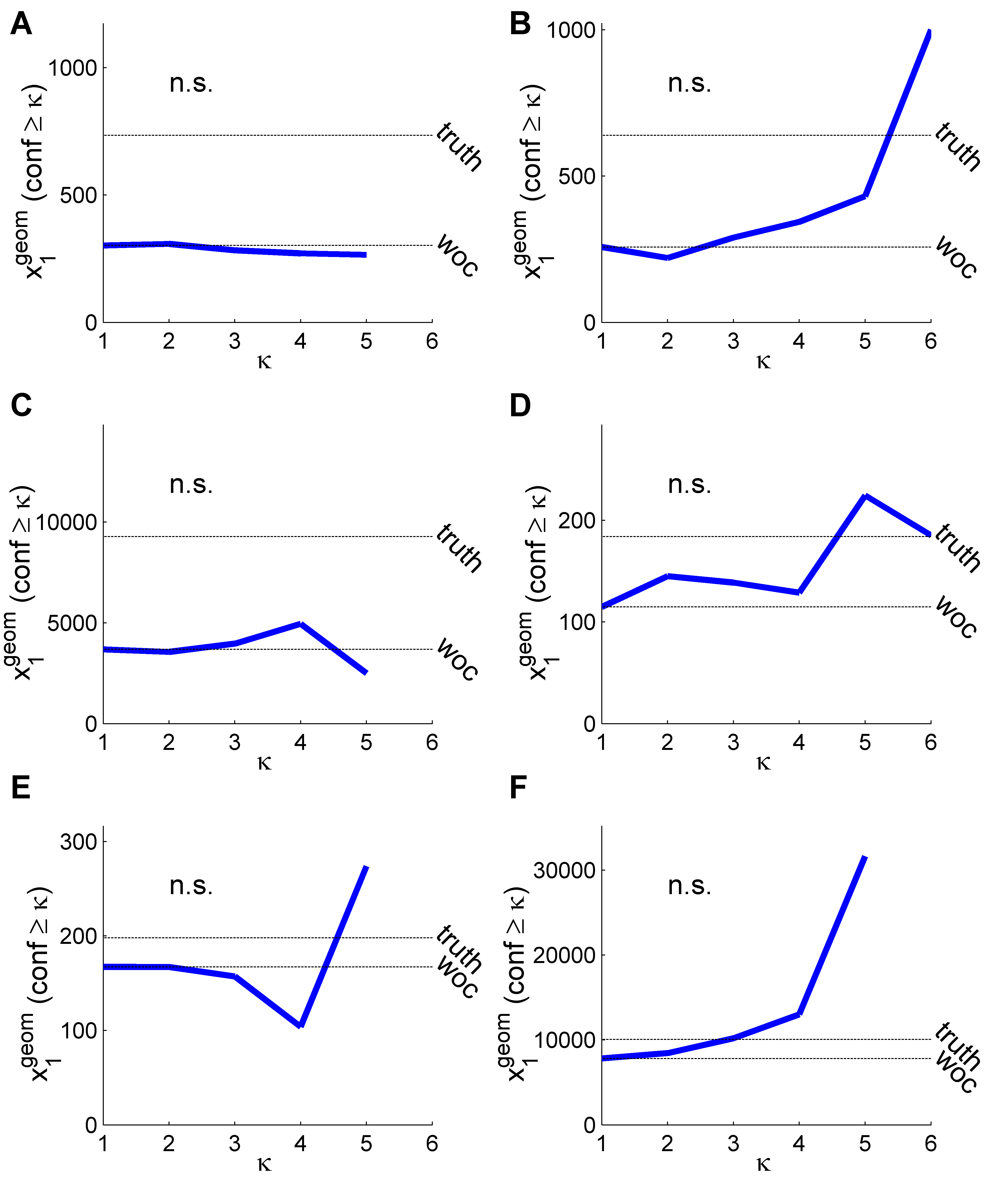

Supplement: S6 Fig — We used a method analogous to that of Figs 2B, 3A, 3C and 3E in main text but for declared confidence instead of social weight. Geometric mean of individuals declaring a value of confidence (conf) in their estimation higher or equal than an integer κ. No value is found to be significant (pmin>0.08, p¯>0.54). The experimental questions are: (A) ‘What is the length of the Swiss/Italian border?’, (B) ‘How many rapes were officially registered in Switzerland in 2006?’, (C) ‘How many assaults were officially registered in Switzerland in 2006?’, (D) ‘What is the population density of Switzerland in inhabitants per square kilometer?’, (E) ‘How many murders were officially registered in Switzerland in 2006?’, and (F) ‘How many more inhabitants did Zurich gain in 2006?’ Data taken from Lorenz et al. [9] (TIFF) [file pcbi.1004594.s006.tiff]
