# Supplementary material for: Improving Collective Estimations Using Resistance to Social Influence
Source: PLoS Comput Biol. 2015 Nov 13;11(11):e1004594. doi: 10.1371/journal.pcbi.1004594 (PMC4643903; doi:10.1371/journal.pcbi.1004594)
Supplement: S1 Text — (DOCX) [file pcbi.1004594.s007.docx]

**Supplementary Text**

***Improving collective estimations using resistance to social influence***

Gabriel Madirolas^1,2^ and Gonzalo G. de Polavieja^1,2^*

^1^Instituto Cajal, Consejo Superior de Investigaciones Científicas, Madrid, Spain

^2^Champalimaud Neuroscience Programme, Champalimaud Center for the Unknown, Lisbon, Portugal

*Corresponding author: [gonzalo.polavieja@neuro.fchampalimaud.org](mailto:gonzalo.polavieja@neuro.fchampalimaud.org)

**Index**

1. **Derivation of Eq 1 in main text**
2. **Derivation that** $\mu_{s}=\mu_{p}$ **in full information condition and** $\mu_{s}=\mu_{p}+\frac{\sigma_{p}^{2}}{2}$ **in aggregated information condition**
3. **Eq 1 in main text for the z-score**
4. **Significance test of whether two questions share the same resisting individuals. Analytical expression.**
5. **Bayesian weights as a simple model of individuality**
6. **Derivation of Eq 1 in main text**

We have shown elsewhere that choices in animal collectives are well described using estimation theory [14, 15]. These models are based on subjects using the probability that $Y$ is the best option, which using Bayes theorem might be written as [14]

|  | $P(Y \text{is} \text{best} \text{option}\vert B,C)\propto P(B\vert Y \text{is} \text{best} \text{option},C)P(Y \text{is} \text{best} \text{option}\vert C)$ |  |
| --- | --- | --- |

where $C$ is the private information the individual has, and $B$ the observed behaviors of the other subjects, specifically how many of them had chosen each of the two options X and Y. The main idea of these models is that animals estimate using both private and social information, and these two sources enter in the estimation as a multiplication.

We now obtain a form of this rule when estimating the value of a continuous variable $x$. The distribution of estimations made by humans is a log-normal [9]. For this reason, we here use the variable $y\equiv\text{log}\left(x\right)$, so a log-normal distribution in $x$ is a normal distribution in$y.$ An estimation that *y* is the correct value based only on private information (*p*) would then be modelled as

|  | . |  |
| --- | --- | --- |

This expression is simply saying that based on private information the individual estimates that the correct value of *y* has a probability centered at $\mu_{p}$ with standard deviation $\sigma_{p}$ .

More generally, individuals make estimations using private and social information. We are here interested in the case in which the social information is made of the estimations made by other individuals, $\vec{y}$ . The estimating individual would then compute the probability that *y* is the correct value given the private information ($p$) and the estimations by others $\vec{y}$, which by Bayes theorem can be expressed as [32]

|  | , |  |
| --- | --- | --- |

where $f_{Y}\left(y \text{is} \text{correct} \text{|}p\right)$ is in Eq S2. The term $f_{\vec{Y}}\left(\left.\vec{y}\right|y \text{is} \text{correct },p\right)$is the probability that the other individuals give the estimations $\vec{y}$ when $y$ is the correct value. It is thus a measure of how reliable the other individuals are. We here consider the cases in which the estimations of the others were given independently of each other as

|  | . |  |
| --- | --- | --- |

The term $n!$ counts all the possible sequences of decisions that lead to the set of values $\vec{y}$, as we are not interested in which particular subject emitted the particular estimation $y_{i}$. We model the terms $f_{Y_{i}}\left(y_{i}\text{|}y \text{is} \text{correct} ,p\right)$ also as Gaussians of the form

|  | . |  |
| --- | --- | --- |

This expression means that, when y is the correct value, individual $i$is modelled as being able to give this value with the highest probability but with standard deviation $\sigma_{s}$. This term thus measures how reliable each individual $i$is, and assumes the same reliability for all individuals and no bias. To add that each of the other individuals has a different reliability we would have a different standard deviation for each individual, $\sigma_{s,i}$. To add a global bias we would have $(y_{i}-y-a)^{2}$ instead of $(y_{i}-y)^{2}$in Eq S5 or individual bias as $(y_{i}-y-a_{i})^{2}$.

Using Eq S5, Eq S4 can be written as

|  | , |  |
| --- | --- | --- |

where

|  | $\mu_{s}\equiv\frac{1}{n}\sum_{i=1}^{n} y_{i}=\text{log}\left(\left[\prod_{i=1}^{n} x_{i}\right]^{1/n}\right)$ |  |
| --- | --- | --- |

is the logarithm of the geometric mean of the estimations made by others, $\text{and}$ $B$ is a term that does not depend on $y$ and that cancels out in the next step. Substituting Eq S6 and Eq S2 into Eq S3, we obtain

|  | , |  |
| --- | --- | --- |

with

|  | $\mu_{f}\equiv\frac{\sigma_{s}^{2}}{\sigma_{s}^{2}+n\sigma_{p}^{2}}\mu_{p}+\frac{n\sigma_{p}^{2}}{\sigma_{s}^{2}+n\sigma_{p}^{2}}\mu_{s}, \sigma_{f}\equiv\frac{\sigma_{s}\sigma_{p}}{\sqrt{\sigma_{s}^{2}+n\sigma_{p}^{2}}}$. |  |
| --- | --- | --- |

A more compact notation is obtained defining a ‘private weight’ $w_{p}$ and ‘social weight’ $w_{s}$ as

|  | $w_{p}\equiv\frac{\sigma_{s}^{2}}{\sigma_{s}^{2}+n\sigma_{p}^{2}}, w_{s}\equiv\frac{n\sigma_{p}^{2}}{\sigma_{s}^{2}+n\sigma_{p}^{2}}$, |  |
| --- | --- | --- |

so the parameters in Eq S9 can be expressed as

|  | $\mu_{f}=w_{p}\mu_{p}+w_{s}\mu_{s}, \sigma_{f}=\sqrt{1-w_{s}} \sigma_{p}$. |  |
| --- | --- | --- |

Introducing Eq S11 into Eq S8, the probability distribution of the logarithm of estimations when subjects combine their private information $p$ and the estimations by the others $\vec{y}$ is written in the compact form

|  |  |  |
| --- | --- | --- |

This is an expression that would model the estimation step for each individual, and from this each individual would produce a concrete value. There are several decision rules individuals could be applying. A simple deterministic rule would simply consist in choosing the value with highest probability. This is however an unlikely rule given the many possible sources of noise, for example memory noise [33]. We will adopt here probabilistic matching, as we have done previously for data in other species [14, 15]. This is a probabilistic rule that does not add additional parameters in the model, according to which the probability of choosing a value *y* is simply the probability that *y* value is the correct one, that is, $f_{Y}\left(y \text{is} \text{correct} \text{|}p,\vec{y}\right)$ in Eq S12.

1. **Derivation that** $\mu_{s}=\mu_{p}$ **in full information condition and** $\mu_{s}=\mu_{p}+\frac{\sigma_{p}^{2}}{2}$ **in aggregated information condition**

We have shown in Eq S7 that the social term $\mu_{s}$reduces to the logarithm of the geometric mean of the estimations made by others, $\mu_{s} =\log\left(x_{s}\right)$, with

|  | $x_{s}=\left(\prod_{i=1}^{n} x_{i}\right)^{1/n}$. |  |
| --- | --- | --- |

But the geometric mean $x_{s}$ is an estimator of the median $\text{exp}\left(\mu_{p}\right)$ of the population [19, 20] , and consequently

|  | $E\left[\mu_{s}\right]=\mu_{p} \to E\left[\mu_{f}\right]=w_{p}\mu_{p}+w_{s}E\left[\mu_{s}\right]=w_{p}\mu_{p}+w_{s}\mu_{p}=\mu_{p}$. |  |
| --- | --- | --- |

making the final distribution in Eq S12 to be centered at $\mu_{p}$. In a second type of experiments that we consider [9], the social information is not the set of estimations made by all other subjects but simply the mean of all of them. When subjects treat this social information in the same way they treat a set of estimations made by other subjects, then $\text{exp}\left(\mu_{s}\right)$ is the arithmetic mean,

|  | $x_{s}=\frac{1}{n}\sum_{i=1}^{n} x_{i}$. |  |
| --- | --- | --- |

For a log-normal distribution, the expected value of the mean is of the form [34]

|  | $E\left[x_{s}\right]=\exp\left(\mu_{p}+\frac{\sigma_{p}^{2}}{2}\right)$. |  |
| --- | --- | --- |

In this case the mean of the final distribution in Eq S12 is

|  | $E\left[\mu_{f}\right]=w_{p}\mu_{p}+w_{s}E\left[\mu_{s}\right]=w_{p}\mu_{p}+w_{s}\left(\mu_{p}+\frac{\sigma_{p}^{2}}{2}\right)=\mu_{p}+w_{s}\frac{\sigma_{p}^{2}}{2}$. |  |
| --- | --- | --- |

We will then use Eq S12 as the distribution of estimations after social interactions with Eq S14 when the social interactions are all the estimations of the other subjects (‘full information’ condition in the main text) and Eq S17 when they receive the mean value of the other subjects (‘aggregated information’ condition).

1. **Eq 1 in main text for the z-score**

We also used in the main text a z-score instead of the variable $y=\text{log}\left(x\right)$ for Fig 1A,B. When the distribution before social interactions is a log-normal with parameters

|  | $\mu_{o}=\mu_{p}, \sigma_{o}= \sigma_{p}$, |  |
| --- | --- | --- |

for the z-score

|  | $z\equiv\frac{\log\left(x\right)-\mu_{p}}{ \sigma_{p}}$, |  |
| --- | --- | --- |

the distribution has parameters

|  | $\mu_{zo}=0, \sigma_{zo}=1$. |  |
| --- | --- | --- |

After social interactions in the ‘full information’ condition the final distribution Eq S12 has the same mean (Eq S14) and a reduced standard deviation

|  | $\mu_{f}=\mu_{p}, \sigma_{f}=\sqrt{1-w_{s}} \sigma_{p}$, |  |
| --- | --- | --- |

that in the z-score gives a distribution with parameters

|  | $\mu_{zf}=0, \sigma_{zf}=\sqrt{1-w_{s}}$. |  |
| --- | --- | --- |

When the social interaction consists in giving the mean (‘aggregated information’ condition) the final distribution Eq S12 has a different mean (Eq S17) and a reduced standard deviation

|  | $\mu_{f}=\mu_{p}+w_{s}\frac{\sigma_{p}^{2}}{2}, \sigma_{f}=\sqrt{1-w_{s}} \sigma_{p}$, |  |
| --- | --- | --- |

that in the z-score corresponds to a Gaussian with parameters

|  | $\mu_{zf}=\frac{\mu_{p}+w_{s}\frac{\sigma_{p}^{2}}{2}-\mu_{p}}{ \sigma_{p}}=w_{s}\frac{ \sigma_{p}}{2} , \sigma_{zf}=\sqrt{1-w_{s}}$. |  |
| --- | --- | --- |

1. **Significance test of whether two questions share the same resisting individuals. Analytical expression.**

We want to compare two selections at random of $N$ and $M$ subjects from a group of $n$ subjects. Both selections are made from the entire original group, so they may have common elements. We are interested in the probability that the two selections have $Z$ or more subjects in common. The probability that in the group of $M$ subjects (the ‘M-group’) you have exactly $Z$ of those in the group of $N$ subjects (the ‘N-group’) is the ratio of the number of favorable cases and all the possible results. The number of favorable cases is given by the product of

$\left(\begin{matrix} N \\ Z \end{matrix}\right)$ : number of combinations of $N$ elements taken in groups of $Z$. Once the N-group is fixed, the above number counts all the possible groups of $Z$ elements that can be extracted from it.

$\left(\begin{matrix} n-N \\ M-Z \end{matrix}\right)$ : number of combinations of the other $n-N$ elements, taken in groups of $M-Z$. This number counts all the possible ways to complete the M-group once the $Z$ common elements are fixed, but without selecting any more elements from the N-group.

The total number of cases is

$\left(\begin{matrix} n \\ M \end{matrix}\right)$ : combinations of $n$ elements taken in groups of $M$. This is the number of all possible M-groups that can be formed from the entire original group.

Then the probability that the two selections have $Z$ subjects in common is

|  | $P\left(Z\right)=\frac{\left(\begin{matrix} N \\ Z \end{matrix}\right)\left(\begin{matrix} n-N \\ M-Z \end{matrix}\right)}{\left(\begin{matrix} n \\ M \end{matrix}\right)}.$ |  |
| --- | --- | --- |

Note that the probability is symmetric under the interchange of $N$ and $M$:

|  | $P\left(Z\right)=\frac{\left(\begin{matrix} M \\ Z \end{matrix}\right)\left(\begin{matrix} n-M \\ N-Z \end{matrix}\right)}{\left(\begin{matrix} n \\ N \end{matrix}\right)}.$ |  |
| --- | --- | --- |

The p-value, defined as the probability of having $Z$ or more common elements is

|  | $p\equiv P\left(\xi\geq Z\right)=\sum_{\xi=Z}^{\text{min}\left(M,N\right)} \frac{\left(\begin{matrix} N \\ \xi\end{matrix}\right)\left(\begin{matrix} n-N \\ M-\xi\end{matrix}\right)}{\left(\begin{matrix} n \\ M \end{matrix}\right)}=\sum_{\xi=Z}^{\text{min}\left(M,N\right)} \frac{\left(\begin{matrix} M \\ \xi\end{matrix}\right)\left(\begin{matrix} n-M \\ N-\xi\end{matrix}\right)}{\left(\begin{matrix} n \\ N \end{matrix}\right)}.$ |  |
| --- | --- | --- |

1. **Bayesian weights as a simple model of individuality**

In this section we derive a model like that of Section 1 but introducing individuality. In section 1, we assumed that the estimating individual models all the other individuals as giving the correct value with the highest probability and with the same standard deviation. In this section we will assume that the standard deviation is different for each individual.

The interest of this approach is that we could in principle use it not only as a model of how human subjects react to social influence, but also as a procedure to get a good prediction for each question in the main text using the individuality of the estimations.

The model results in a prediction given by the weighted average of the estimations of the population, with each individual weighting more the lower the social weight (Eq S31 below). This result is intuitive with individuals having more importance in the prediction the less they are influenced by social information.

However, we also show in this section that this approach does not give good predictions. The reason for this failure was expected as we are only modelling individuality in the standard deviation. We are still assuming in this model that all individuals can give the correct value with the highest probability. The experimental data indicates this is not the case as different individuals or subpopulations can have different biases from the truth (for example, see Fig 2A in main text). Modelling individuality in the bias means that each individual can have the highest probability of giving an estimation shifted from the correct value. This can be modelled formally, but as bias is defined respect to truth, the model would have no predictive value. Instead, our proposal is to follow the procedure of the main text, which uses only individuals with low social weight as they are on average closer to correct values. Alternatively, one may consider the methods in the main text as a strong prior and include the weights we obtain here of the individuals extracted.

The derivation of this model is as follows. We introduce individuality by considering that instead of all individuals having the same reliability as in Eq S6, each individual has a different value of reliability as

|  | , |  |
| --- | --- | --- |

with individuals with smaller standard deviation $\sigma_{s,i}$ more reliably giving an estimation closer to the correct value, and we can use the properties of the product of Gaussians [35] to write

|  | $\mu_{s}=\frac{\sum_{i=1}^{n} \left(y_{i}/\sigma_{s,i}^{2}\right)}{\sum_{i=1}^{n} \left(1/\sigma_{s,i}^{2}\right)}, 1/\sigma_{s}^{2}=\sum_{i=1}^{n} \left(1/\sigma_{s,i}^{2}\right)$. |  |
| --- | --- | --- |

We can estimate the individual standard deviations $\sigma_{s,i}$ in the following way. The $\sigma_{s,i}$ measures the width of the probability distribution for each subject, a role played by $\sigma_{p}$ in Eq S2. Similarly to that case, we can use how each subject reacts to social influence, Eq S11, to obtain how standard deviation relates to the social weight as

|  | $\sigma_{s,i}=\frac{\sigma_{f}}{\sqrt{1-w_{s,i}}}$, |  |
| --- | --- | --- |

with $w_{s,i}$ the experimental social weight. We assume that$\sigma_{f}$ is the same for all subjects as an approximation we need to take with the data at hand. The specific value of $\sigma_{f}$ is not important as it cancels out in the next steps. Using S30, we can express S29 as

|  | $\mu_{s}=\frac{\sum_{i=1}^{n} \left(\frac{1-w_{s,i}}{\sigma_{f}^{2}}y_{i}\right)}{\sum_{i=1}^{n} \left(\frac{1-w_{s,i}}{\sigma_{f}^{2}}\right)}=\frac{\sum_{i=1}^{n} \left(\left(1-w_{s,i}\right)y_{i}\right)}{\sum_{i=1}^{n} \left(1-w_{s,i}\right)}$. |  |
| --- | --- | --- |

Expression S31 gives the prediction of this model as a weighted average of the logarithm of estimations, with weights given by $1-w_{s,i}$, and $w_{s,i}$ the social weights obtained from data. Those individuals that are influenced more by social information weight less in the prediction, as expected.

We compared the prediction of S31 with the correct values for the 6 experimental questions in [9]. The experimental values $w_{s,i}$ can be below 0 and above 1 unlike those of the theory, so we make the comparison eliminating these cases in different ways, as in the table below. Irrespective of the method, the comparison of prediction and correct values is poor.

| Question | truth | WOC | ignore |w_s_|>1 | ignore w_s_>1, and collapse w_s_<0 to 0 | ignore |w_s_|>1 and |w_s_|<0 |
| --- | --- | --- | --- | --- | --- |
| Border | 734 | 302  (-59%) | 397  (-46%) | 389  (-47%) | 333  (-55%) |
| Rapes | 639 | 257  (-60%) | 241  (-62%) | 256  (-60%) | 244  (-62%) |
| Assaults | 9272 | 3685  (-60%) | 4834  (-48%) | 4823  (-48%) | 4412  (-52%) |
| Population | 184 | 115  (-38%) | 110  (-40%) | 116  (-37%) | 100  (-46%) |
| Murders | 198 | 167  (-16%) | 149  (-25%) | 153  (-23%) | 139  (-30%) |
| Immigrants | 10067 | 7819  (-22%) | 8414  (-16%) | 9224  (-8.4%) | 7666  (-24%) |
|  | **Average** | -42% | -40% | -37% | -45% |

**Table. Prediction of the model derived in this section.** **Border**, ‘What is length of the Swiss/Italian border?’; **Rapes**, ‘How many rapes were officially registered in Switzerland in 2006?’; **Assaults**, ‘How many assaults were officially registered in Switzerland in 2006?’; **Population**, ‘What is the population density of Switzerland in inhabitants per square kilometer?’; **Murders**, ‘How many murders were officially registered in Switzerland in 2006?’; **Immigrants,** ‘How many more inhabitants did Zurich gain in 2006?

These poor predictions were expected as the model only takes into account individuality in the standard deviation of estimations for each individual and not in the biases respect to truth that individuals can have.

**References**

Papoulis A, Pillai SU (2002) Probability, Random Variables, and Stochastic Processes. 103. Tata McGraw-Hill Education.

Vul E, Pashler H (2008) Measuring the crowd within probabilistic representations within individuals. Psychol. Sci. 19(7), 645-647.

Johnson NL, Kotz S, Balakrishnan N (1994) Continuous Univariate Distributions 1, 211-212. John Wiley & Sons.

Bromiley PA (2003) Products and convolutions of Gaussian probability density functions. Tina-Vision Memo, vol. 3. Available: <http://tina.wiau.man.ac.uk/docs/memos/2003-003.pdf>
