# Supplementary material for: Improving Collective Estimations Using Resistance to Social Influence
Source: PLoS Comput Biol. 2015 Nov 13;11(11):e1004594. doi: 10.1371/journal.pcbi.1004594 (PMC4643903; doi:10.1371/journal.pcbi.1004594)
Supplement: S1 Table — Summary of the results of the significance tests in main text. Kolmogorov-Smirnov tests were run with Matlab to check normality. Permutations method were performed as explained in the main text (Methods) to test for the equality of means and equality of variances. For the no difference of means, two sample t-tests were run with Matlab to check compatibility with permutations method. For the no difference of variances, two sample F-tests were run with Matlab with the same purpose. No discrepancies in the acceptance/rejection of the null hypothesis were found in any of the no difference tests. Bayesian tests are based on the likelihood of the experimental data given a certain value of the parameters. More specifically, we follow the reference [21] in the main text: Kruschke JK (2013) Bayesian estimation supersedes the t test. J. Exp. Psychol. Gen. 142(2), 573. The method generates a probability distribution of the most credible values of the parameters (or their difference for two distribution comparison). If a value falls outside the 95% highest density interval (HDI) then it is not considered to be a credible value of the parameter or difference of parameters. For the distribution to be considered credibly normal, a value for the degrees of freedom parameter of log10(v) > log10(30)≈1.48 is required. Only one discrepancy was found with the null hypothesis methods, and the Bayesian test cannot accept the normality of the estimation distribution generated in the second trial of the ‘aggregated information’ condition. Although the Kolmogorov-Smirnov test did not reject the normality hypothesis, the p-value was slightly above 0.05. In the main text and in S1 Fig this poor value is explained by the fact that the distribution is better explained the sum of 24 Gaussians with very similar parameters. (DOCX) [file pcbi.1004594.s008.docx]

| Property | Samples  (information condition, trial) | Null Hypothesis Significance Tests  (*p*-value, accept) | Bayesian Tests  (95% HDI, accept) |
| --- | --- | --- | --- |
|  |  | **Kolmogorov-Smirnov** |  |
| Normality | no info, t1 | 0.41, yes | (0.896, 2.04), yes |
|  | no info, t2 | 0.27, yes | (0.841, 2.01), yes |
|  | full info, t1 | 0.36, yes | (0.871, 2.05), yes |
|  | full info, t2 | 0.33, yes | (0.568, 1.51), yes |
|  | agg. info, t1 | 0.95, yes | (1.07, 2.11), yes |
|  | agg. info, t2 | 0.0501, yes | (0.384, 0.828), no |
|  |  | **Permutations** |  |
| Equality of means | no info, t1 - no info, t2 | 0.75, yes | (-0.138, 0.196), yes |
|  | full info, t1 - full info, t2 | 0.14, yes | (-0.256, 0.0205), yes |
|  | agg. info, t1 - agg. info, t2 | <10e-6, no | (-0.551, -0.281), no |
| Equality of variances | no info, t1 - no info, t2 | 0.62, yes | (-0.151, 0.0905), yes |
|  | full info, t1 - full info, t2 | <10e-6, no | (0.203, 0.411), no |
|  | agg. info, t1 - agg. info, t2 | <10e-6, no | (0.271, 0.475), no |
|  | full info, t2 - agg. info, t2 | 0.48, yes | (-0.139, 0.0164), yes |

**S1 Table. Kolmogorov-Smirnov, Permutations and Bayesian Significance Tests**

Summary of the results of the significance tests in main text. **Kolmogorov-Smirnov** tests were run with Matlab to check normality. **Permutations** method was performed as explained in the main text (Methods) to test for the equality of means and equality of variances. For the no difference of means, two sample t-tests were run with Matlab to check compatibility with permutations method. For the no difference of variances, two sample F-tests were run with Matlab with the same purpose. No discrepancies in the acceptance/rejection of the null hypothesis were found in any of the no difference tests. **Bayesian tests** are based on the likelihood of the experimental data given a certain value of the parameters. More specifically, we follow the reference 21 in the main text: Kruschke JK (2013) Bayesian estimation supersedes the t test. J. Exp. Psychol. Gen. 142(2), 573. The method generates a probability distribution of the most credible values of the parameters (or their difference for two distribution comparison) is generated. If a value falls outside the 95% highest density interval (HDI) then it not considered to be a credible value of the parameter or difference of parameters. For the distribution to be considered credibly normal, a value for the degrees of freedom parameter of log_10_($v$) > log_10_(30)$\approx$ 1.48 is required. Only one discrepancy was found with the null hypothesis methods, and the Bayesian test cannot accept the normality of the estimation distribution generated in the second trial of the ‘aggregated information’ condition. Although the Kolmogorov-Smirnov test did not reject the normality hypothesis, the p-value was slightly above 0.05. In the main text and in S1 Fig this poor value is explained by the fact that the distribution is better explained the sum of 24 Gaussians with very similar parameters.
